# A Blood-Based Immune Gene Signature with Prognostic Significance in Localized Prostate Cancer

**DOI:** 10.3390/cancers15143697

**Published:** 2023-07-20

**Authors:** Sotirios P. Fortis, Panagiota Batsaki, Savvas Stokidis, Dimitra Moschandreou, Elisavet Grouzi, Constantin N. Baxevanis, Angelos D. Gritzapis, Maria Goulielmaki

**Affiliations:** 1 Cancer Immunology and Immunotherapy Center, Cancer Research Center, Saint Savas Cancer Hospital, 11522 Athens, Greece; fortis@ciic.gr (S.P.F.); pmpatsaki@agsavvas-hosp.gr (P.B.); savstok@gmail.com (S.S.); costas.baxevanis@gmail.com (C.N.B.); mgoulielmaki@ciic.gr (M.G.); 2 Department of Transfusion Service and Clinical Hemostasis, Saint Savas Cancer Hospital, 11522 Athens, Greece; dimitramos@gmail.com (D.M.); grouzielisavet@gmail.com (E.G.)

**Keywords:** prostate cancer, prognostic biomarker, gene expression, eight-gene signature, the cancer genome atlas, pan-cancer

## Abstract

**Simple Summary:**

The discovery of prognostic biomarkers constitutes an important issue because it allows the tailoring of therapeutic treatments, thus avoiding over-treatment and side effects. In this context, reliable prognostic biomarkers advance the field of precision oncology. Such biomarkers are usually discovered in the tumor tissue, which is not an easy task given the inaccessibility of malignant tissue in many types of cancer. In this work, we could identify a prognostic signature consisting of eight genes for prostate cancer patients. This biosignature was identified in peripheral blood samples, which are easy to access and, importantly, have a significant prognostic value for various types of cancer.

**Abstract:**

Prostate cancer (PCa) is one of the most common male cancers worldwide and one of the deadliest if unsuccessfully treated. Τhe need for reliable, easily accessible immune-related molecular biomarkers that could be combined with clinically defined criteria, including PSA and Gleason score, to accurately predict PCa patients’ clinical outcomes is emerging. Herein, we describe for the first time a blood-identified immune-related gene signature comprising eight upregulated multi-functional genes associated with poor prognosis. Next-generation sequencing (NGS) analysis of PCa patients’ peripheral blood samples revealed a more than three-fold upregulation of each of the eight genes as compared to samples originating from healthy donors. The construction of gene and protein interaction networks revealed different extents of the functional implications of these genes in the regulation of cell proliferation and immune responses. Analysis of the available data from *The Cancer Genome Atlas* (TCGA) regarding gene expression and survival of prostate adenocarcinoma (PRAD) and pan-cancer (PANCAN) patients revealed that intra-tumoral upregulation of this eight-gene signature (8-GS) was associated with poor 5-year progression-free intervals in PCa patients, even in those with high Gleason scores, and also with an unfavorable prognosis for cancer patients irrespective of the cancer type and even in the early stages. These observations suggest that further investigation of the 8-GS prospectively in randomized clinical trials, in which clinical benefit in terms of evaluating time to disease progression can be assessed, is warranted.

## 1. Introduction

Prostate cancer (PCa) is the most commonly occurring and one of the deadliest male malignancy worldwide and pertains mainly to advanced-aged individuals [1]. The clinical presentation of the disease varies from localized to advanced PCa, which may culminate in a rapidly progressing metastatic disease [2]. The overall prognosis for patients with localized or regional PCa under standard treatments is among the best of all cancer types, with the 5-year relative survival rate reaching more than 99% in these cases [3]. Nevertheless, the annual PCa mortality rate is still considerably elevated due to the high incidence of the disease.

The diagnosis of PCa is based on established clinical practices, including primarily tumor tissue biopsy, prostate-specific antigen (PSA) testing, digital rectal examination, and magnetic resonance imaging. Gene expression signatures, or expression levels of isolated genes, have come to the forefront as a sensitive and specific biomarker for the detection and/or prognosis of PCa patients [4]. However, the expression levels of most of these biomarkers are measured in the patients’ biopsied material, which alone is a limiting factor due to the difficulty of sample collection [5,6]. On the other hand, commercially available urine-based biomarkers, including PCA3, TMPRSS2, HOXC6, etc., lack either sensitivity or specificity [7]. Although cancer development is characterized by multiple genetic and epigenetic alterations as well as aberrant gene expression, the exact molecular events that contribute to cancer progression, including PCa, are still not fully explored. The combined assessment of certain clinically established criteria, such as PSA and the Gleason score, alongside molecular markers, including driver mutations and gene expression signatures, has been highlighted as putative biomarkers for PCa risk stratification and prognosis [8].

Regarding localized PCa, contemporary models for appraising the risk stratification post-local therapy depend on PSA levels, the International Society of Urological Pathology (ISUP) grade on biopsies, and the T stage [9]. However, these biomarkers lack sufficient specificity and sensitivity for the diagnosis of PCa at early stages [10,11,12]. Hence, there is an urgent need for the identification of reliable biomarkers that could function as predictors of clinical outcomes in patients with early-stage PCa. Currently, a few alternative prognostic biomarkers for early-stage PCa patients have been described [9,13,14]. We have previously described a gene signature composed of six immune-related genes with possible predictive potential in radiotherapy-treated PCa patients [15]. In the present study, by implementing a high-throughput quantitative analysis, we sought to identify differences in immune-related gene expression in the peripheral blood of PCa patients with localized disease compared to age-matched healthy donors. In doing so, we could identify eight genes in the periphery that were upregulated in PCa patients as compared to healthy donors. Given the slow progression of early-stage PCa, we verified the prognostic value of these expressed genes, which were also extracted from the prostatic tissue of PCa patients at all stages of the disease, whose data are available from The Cancer Genome Atlas (TCGA) database. In this way, novel immune-based biomarkers with expression levels that coincide between the TME and the peripheral blood may be proposed as prognosticators in PCa patients.

## 2. Materials and Methods

### 2.1. Selection of Study Individuals and Sample Collection

A total of 23 patients who were diagnosed with adenocarcinoma of the prostate between January 2019 and June 2020 were retrospectively recruited for the present study. All patients were under androgen deprivation therapy (ADT) at the time of enrollment. Six patients additionally underwent radical prostatectomy (RP) before sample collection. Due to the different clinical characteristics and treatment options, we sought to have a group of patients as homogeneous as possible. Patients who had already received ADT but had not started radiotherapy were eligible for our study. The clinicopathological features of the PCa patients are presented in Table 1. Seventeen age-matched, healthy male volunteers were also included in the study. Peripheral blood was collected from PCa patients and healthy donors directly into K2-EDTA tubes (BD, Mississauga, ON, Canada) and was immediately transferred to the laboratory for subsequent isolations.

### 2.2. Ethics Approval

This study complied with the ethical principles of the Declaration of Helsinki and was approved by the ethics committees of the Saint Savas Cancer Hospital (approval no. IRB-ID6777/14-06-2017). In accordance with the institutional guidelines, men signed an institutional review board-approved, protocol-specific informed consent form permitting the prospective collection of peripheral blood.

### 2.3. RNA Isolation

Following peripheral blood collection, total RNA was extracted from each sample with the PureLink^TM^ Total RNA Blood Kit (Invitrogen, Thermofisher, Waltham, MA, USA), according to the manufacturer’s instructions. Accordingly, the extracted RNA was incubated with the ezDNase^TM^ Enzyme (Invitrogen, Thermofisher, Waltham, MA, USA) for 2 min at 37 °C to ensure digestion of any remaining gDNA. The extracted RNA was quantified using the Qubit^TM^ RNA HS Assay Kit (Thermofisher, Waltham, MA, USA) on a Qubit Fluorometer 3.0 (Thermofisher, Waltham, MA, USA) and stored at −80 °C.

### 2.4. Library Preparation and Next-Generation Sequencing

The oncomine immune response research assay (OIRRA) (Thermofisher, Waltham, MA, USA) was used for the quantification of the expression levels of immune response genes. The panel enables the simultaneous assessment of 398 genes related to variable immune-system functions, including immune-cell adhesion and migration, T-cell receptor co-expression, immune checkpoints, cytokine signaling, lymphocyte infiltration, and immune-cell markers. A total RNA input of 10 ng was used for library preparation, following the manufacturer’s recommendations. Briefly, reverse transcription was performed using the SuperScript^TM^ VILO^TM^ cDNA Synthesis Kit (Thermofisher, Waltham, MA, USA). For target amplification and subsequent library preparation, the Ion AmpliSeq^TM^ Library Kit 2.0 (Ion Torrent^TM^, Thermofisher, Waltham, MA, USA) and the IonXpress^TM^ Barcode Adapters (Ion Torrent^TM^, Thermofisher, Waltham, MA, USA) were used, respectively. The amplified libraries were purified using the Agencourt^TM^ AMPure^TM^ XP Reagent (BeckmanCoulter Life Sciences, Brea, CA, USA) and were separately quantified by qPCR using the Ion Library TaqMan^TM^ Quantitation Kit (Ion Torrent^TM^, Thermofisher, Waltham, MA, USA) on the Quantstudio 5.0 (Thermofisher, Waltham, MA, USA). Accordingly, the libraries were diluted to 50 pM of individual concentration and pooled. After template preparation and library loading on an Ion 530^TM^ Chip using the Ion Chef^TM^ System (single-end sequencing, 400 bp nucleotide length), sequencing took place on the Ion GeneStudio^TM^ S5 System (all from Ion Torrent^TM^, Thermofisher, Waltham, MA, USA).

### 2.5. Gene Expression Analysis from Blood Samples

The immune response-related gene expression analysis was performed using the Ion Reporter^TM^ Software 5.16 equipped with the ion torrent immune response RNA plugin for the generation of gene transcript data. Specifically, the reads per million (RPM) values were log-transformed and normalized, and the gene expression values were further analyzed on the Affymetrix transcriptome analysis console (TAC) 4.0 software. The minimal read cutoff was set to 1.5 million reads per sample. Gene expression levels were expressed as normalized average log2 values and were subsequently transferred to GraphPad Prism 9.3.1 for Windows (GraphPad Software, Inc., San Diego, CA, USA), along with the resulting fold change (FC) values as compared to their expression in healthy donors. In total, 77 genes were found differentially regulated in the peripheral blood of healthy donors vs. PCa patients; 40 genes were found upregulated and 37 genes were found downregulated. Only genes that were at least 3-fold upregulated (*n* = 22) in PCa patients compared to healthy donors were included in the subsequent analyses. In turn, we used the TCGA-PRAD database in order to investigate how many of these genes were upregulated in PCa tissue compared to adjacent normal tissue (designated as solid tissue normal) and whose concomitant upregulation was significantly associated with 5-year PFI in PCa patients. In this way, we ended up with the proposed eight genes. Multiple Mann–Whitney (unpaired) tests were implemented for the identification of differential gene expression between healthy subjects and PCa patients. The data are graphed as the mean with a 95% confidence interval. A correlation matrix analysis using the Pearson coefficient was adopted for the joint assessment of the correlation between the eight genes that comprise the proposed signature in PCa patients compared to healthy donors. *p* values lower than 0.05 were considered statistically significant. The interaction network of the genes comprising the 8-GS was constructed using the GeneMANIA database [16]. In turn, the respective interprotein network was built via the STRING database [17]. Further functional analysis was performed using the gene set enrichment analysis (GSEA) 4.3.2 software and the human molecular signatures database (MSigDB) 2023.1 [18,19].

### 2.6. TCGA Data Analysis

The gene expression and matched survival data of prostate adenocarcinoma (PRAD) and pan-cancer (PANCAN) patient cohorts that are available from The Cancer Genome Atlas (TCGA) database were collected using the Xena browser (http://xena.ucsc.edu; accessed on 16 May 2023). Correlation analyses were performed using the mRNA expression levels of genes of interest, progression-free interval (PFI), overall survival (OS), PFI-time, OS-time, and phenotypic data (sample type and gleason score for PRAD; Sample type and clinical stage for PANCAN). Only primary tumor samples (PRAD, *n* = 497; PANCAN, *n* = 9636) were used after the removal of samples that were missing the required data (samples with nulls). The median value was used for patient classification into low and high gene expression groups (below and above the equal median, respectively). For survival analysis based on the expression of the 8-GS, high or low expressors were considered those patients whose tumors expressed all 8 genes above (equal) or below the median, respectively. Survival data were transferred to GraphPad Prism 8.0.2 for Windows (GraphPad Software, Inc., San Diego, CA, USA), and Kaplan-Mayer curves were plotted. Statistical significance was assessed using both the log rank and the Gehan-Breslow method, with *p* values lower than 0.05 being considered statistically significant.

## 3. Results

### 3.1. Demographics of the Study Cohorts

The enrolled patients’ characteristics are presented in Table 1. The mean age of the patients was 73 years (range: 53–81). 15 patients (65.2%) had strictly contained disease (T1/T2 stage), while 8 patients (34.8%) were diagnosed with T3-staged tumors. Gleason score (GS) ranged between 6 and 9, and baseline prostate-specific antigen (PSA) levels ranged between 5.51 and 100.00 ng/mL (mean 18.74 ng/mL). All patients have been receiving ADT for the last three months before blood sampling. Six patients had RP prior to ADT.

### 3.2. Differential Gene Expression in the Peripheral Blood of PCa Patients Compared to Healthy Donors

**Figure 1:**
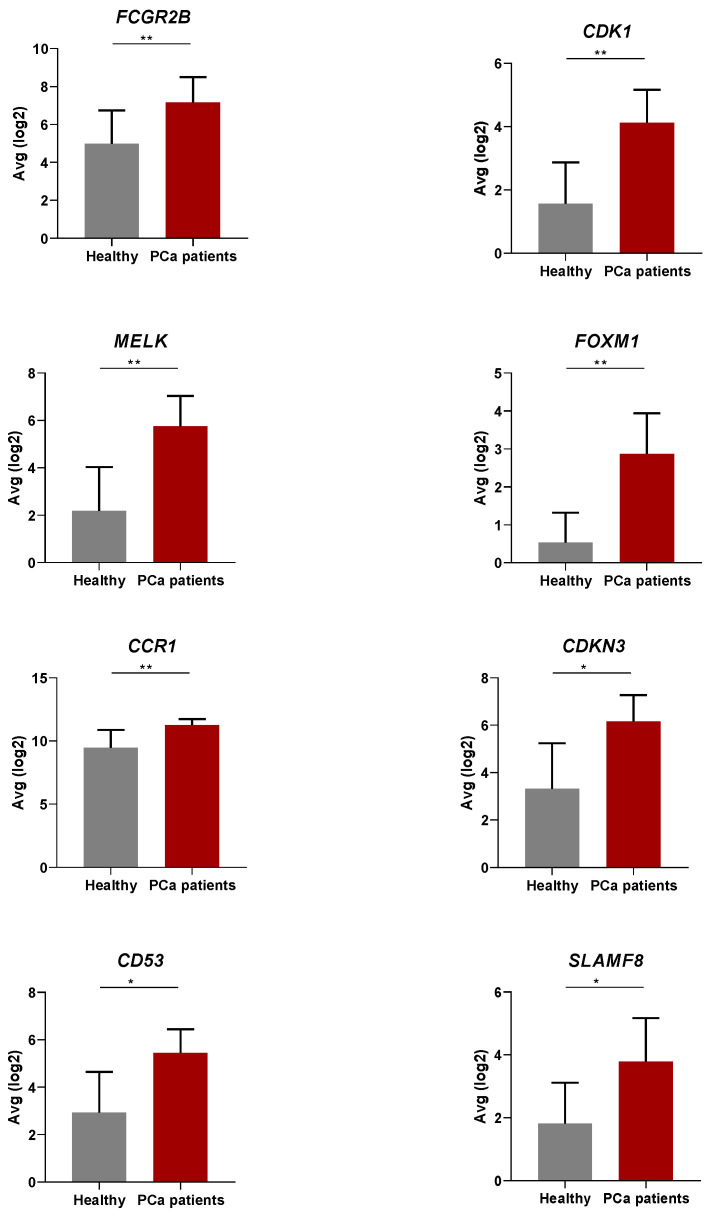
Expression levels of the genes comprising the eight-gene signature (8-GS) in the peripheral blood of 23 prostate cancer (PCa) patients compared to 17 healthy individuals. Each graph corresponds to the expression levels of each single gene in healthy controls and PCa patients. Each column shows the log2-transformed average mRNA levels of each gene normalized by Reads Per Million (RPM). Statistically significant differences between gene expression in the blood of PCa patients and healthy controls were identified by performing individual non-parametric Mann-Whitney (unpaired) tests. The error bars designate the average (Avg) values with a 95% confidence interval range. *p*-values below 0.05 signify statistical significance. *, *p* < 0.05; **, *p* < 0.01.

Analysis of the immune response gene expression profiles revealed eight upregulated genes with an FC > 3 as compared to healthy donors (*FCGR2B*, *p* = 0.0056; *CDK1*, *p* = 0.0046; *MELK*, *p* = 0.0056; *FOXM1*, *p* = 0.0022; *CCR1*, *p* = 0.0054; *CDKN3*, *p* = 0.0127; *CD53*, *p* = 0.0259; *SLAMF8*, *p* = 0.0157) (Figure 1).

**Figure 2:**
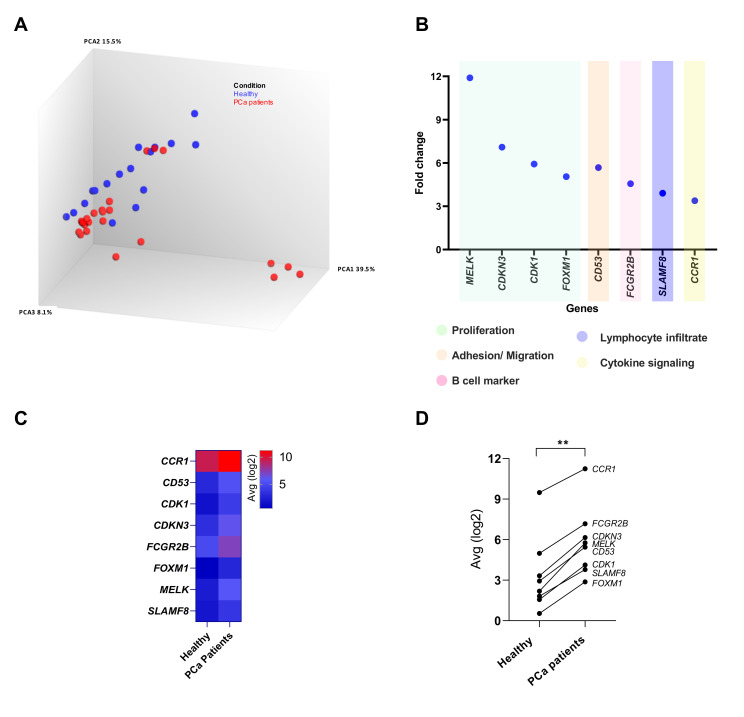
Variations in gene expression of the eight-gene signature (8-GS) in the peripheral blood of 23 prostate cancer (PCa) patients and 17 healthy individuals. (**A**) Principal component analysis (PCA) plot of the RNA-seq results of the PCa patients and healthy donors based on the expression levels of the 398 genes of the Oncomine Immune Response Research Assay (OIRRA). (**B**) Classification of the genes comprising the 8-GS into functional annotation groups. Four genes are involved in cell proliferation pathways, while the remaining genes mediate pathways related to adhesion/migration, B-cells, lymphocyte infiltration, and cytokine signaling. (**C**) Heatmap of the range of expression levels of the eight genes in healthy donors vs. PCa patients. For the analysis, the median expression levels of each gene were used for data visualization. Different colors correspond to different gene expression levels, ranging from red (high expression, max. value 11.24) to blue (low expression, min. value 0.53). (**D**) The joint assessment of the 8-GS shows a statistically significant upregulation (*p* = 0.0011) in the peripheral blood of PCa patients compared to healthy controls. The depicted lines represent the conversion of the corresponding gene from lower (healthy controls) to higher expression levels (PCa patients). **, *p* < 0.01.

The variability of the gene expression data between healthy donors and PCa patients is depicted in the principal component analysis (PCA) plot in Figure 2A. Half of the upregulated genes (*MELK*, *CDKN3*, *CDK1*, and *FOXM1*) are associated with cell proliferation functions; these four genes showed more than 5-fold upregulation (Figure 2B). Alongside, *CD53*, which is related to cell adhesion/migration properties, was upregulated by 5.7 folds, whereas the three remaining genes, namely *FCGR2B, SLAMF8,* and *CCR1*, had a 4.6-, 3.9-, and 3.4-fold increase in gene expression, respectively (Figure 2B); these genes are implicated in B-cell functions, lymphocyte infiltration, and cytokine signaling, respectively (Figure 2B). The differential gene expression levels in the blood of healthy donors and PCa patients are also presented in the form of a heatmap (Figure 2C). Correlation analysis of the joint assessment of the 8 genes as a signature signified a statistically significant higher expression in PCa patients vs. healthy controls (*p* < 0.01, r = 0.9524; Figure 2D).

### 3.3. High Expression of the 8 Genes in Primary Tumor Tissue Associates with a Lower PFI in PCa Patients

**Figure 3:**
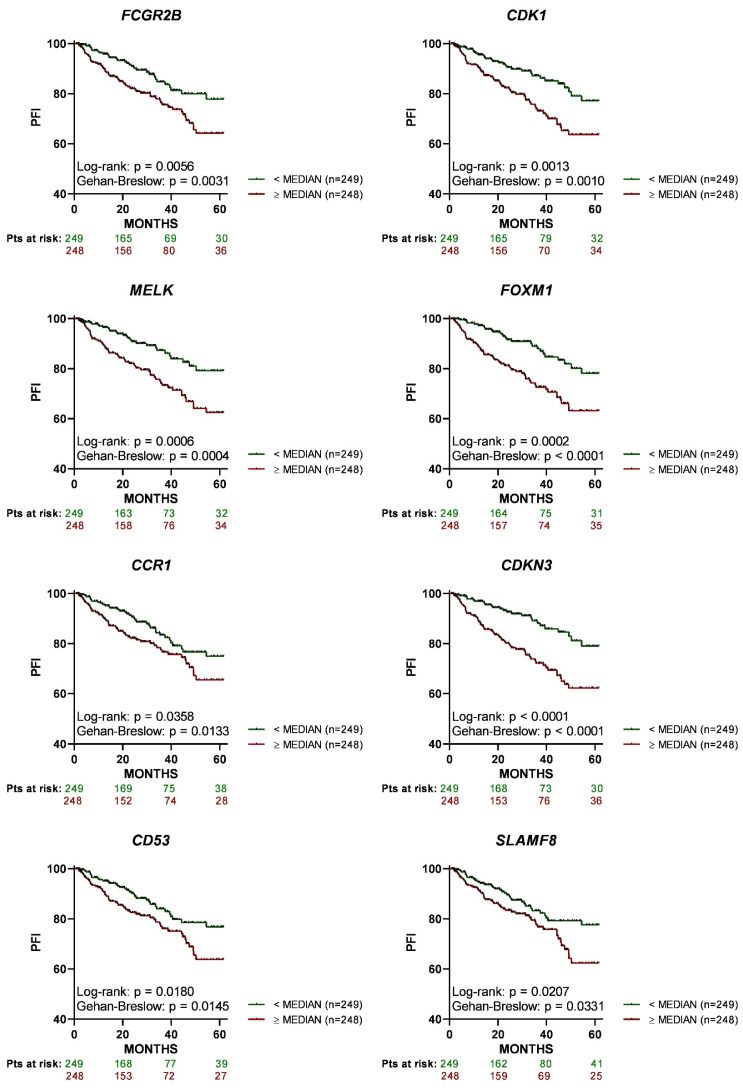
All of the upregulated genes that constitute the eight-gene signature (8-GS) are associated with a statistically significant worse progression-free interval (PFI) in prostate cancer (PCa) patients. Kaplan-Meier curves for the 5-year PFI were graphed based on the dichotomized median expression of each gene of the 8-GS in the tumor tissue of PCa patients, based on data extracted from the PRAD database. PCa patients with high expression of each gene (red lines; above or equal to median) had a significantly worse PFI as compared to patients with low expression of each gene (green lines; below median). Pts, patients.

Using the PRAD dataset, we also examined the association between gene expression and PFI for each of the eight genes separately. The results depicted in Figure 3 show that PCa patients whose tumors expressed each one of these genes separately at high levels (i.e., above/equal median) had a significantly shorter PFI as compared to patients with low gene expression (i.e., below median).

**Figure 4:**
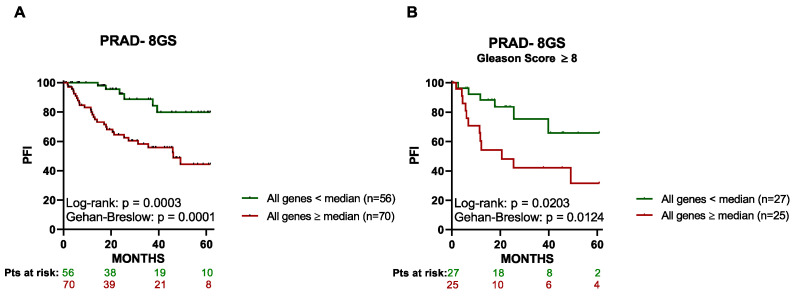
High expression of the eight-gene signature (8-GS) in the tumor tissue is associated with a lower progression-free interval (PFI) in prostate cancer (PCa) patients. Kaplan–Meier curves for the 5-year PFI in PCa patients were designed based on the dichotomized median expression of the 8-GS in prostate tumor tissue. (**A**) Association between the expression levels of the 8-GS and 5-year PFI of PCa patients. Only patients with all genes above (or equal to) their respective median expression value and patients with all genes below their respective median expression value (*n* = 126) are included in the graph. (**B**) Association between the expression levels of the 8-GS and 5-year PFI of PCa patients with a Gleason Score above 8. Only patients with all genes above (or equal to) their respective median expression value and patients with all genes below their respective median expression value (*n* = 52) are included in the graph. The data were extracted from the PRAD dataset. The red lines indicate patients with high expression (expression value ≥ median), while the green lines indicate patients with low expression (expression value < median) of the 8-GS. Pts, patients.

Importantly, we observed a similar pattern for PFI when the 8 genes were analyzed jointly as a signature (8-GS). Thus, as shown in Figure 4A, patients with primary tumors expressing all of the eight genes above or equal to their respective median values (*n* = 70) had a significantly worse 5-year PFI compared to those expressing the above 8-GS below the median (*n* = 56). The same pattern was legible when subcategorization of the patient cohort by Gleason Score was performed; patients with a high Gleason score (≥8) and low expression of the 8-GS (*n* = 27) had a significantly better 5-year PFI compared with patients with a Gleason score ≥8 but high gene expression (*n* = 25; Figure 4B). Interestingly, the investigation into the possible correlation between the expression of the eight identified genes in the peripheral blood of PCa patients and the Gleason score showed no statistically significant difference between patients with high (≥8) and low (<8) Gleason scores (Figure S1). 

**Figure 5:**
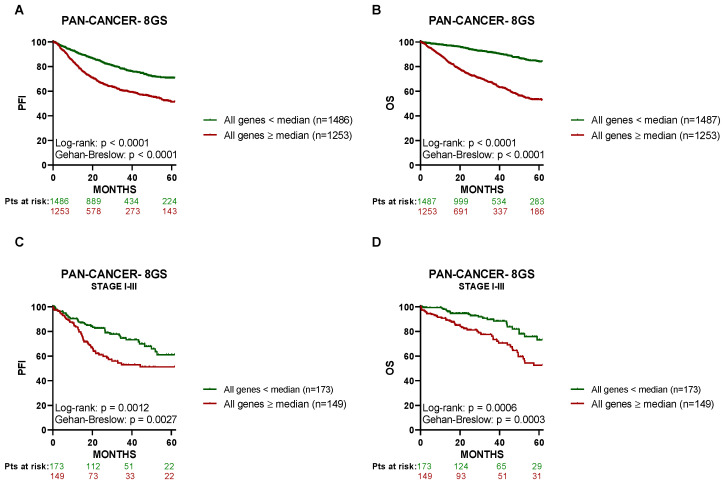
High expression levels of the eight-gene signature (8-GS) are correlated with worse progression-free and overall survival rates in cancer patients, regardless of the cancer type. Kaplan–Meier curves for the 5-year progression-free interval (PFI) in pan-cancer patients were designed based on the dichotomized median expression of the 8-GS in pan-cancer tissue. (**A**) Association between the expression levels of the 8-GS and 5-year PFI of pan-cancer patients. Only patients with all genes above (or equal to) their respective median expression value and patients with all genes below their respective median expression value (*n* = 2739) are included in the graph. (**B**) Association between the expression levels of the 8-GS and 5-year OS of pan-cancer patients. Only patients with all genes above (or equal to) their respective median expression value and patients with all genes below their respective median expression value (*n* = 2740) are included in the graph. (**C**) Association between the expression levels of the 8-GS and 5-year PFI of pan-cancer patients with stage I–III disease. Only patients with all genes above (or equal to) their respective median expression value and patients with all genes below their respective median expression value (*n* = 322) are included in the graph. (**D**) Association between the expression levels of the 8-GS and 5-year OS of pan-cancer patients with stage I–III disease. Only patients with all genes above (or equal to) their respective median expression value and patients with all genes below their respective median expression value (*n* = 322) are included in the graph. Pts, patients.

Since the identified 8-GS demonstrated a meaningful correlation with the PFI of PCa patients, we then sought to investigate its potential prognostic value across all of the available TCGA patients’ samples, regardless of the cancer type. As shown in Figure 5, cancer patients expressing the 8-GS above or equal to their respective median value had a significantly lower 5-year PFI (*n* = 1486; Figure 5A) and a further diminished 5-year OS (*n* = 1487; Figure 5B) compared to those expressing the 8-GS below the median (*n* = 1253). Notably, when the investigation cohort was limited only to confirmed stage I to III cancer patients (*n* = 322), similar PFI and OS patterns were detected (Figure 5C,D, respectively). In regard to patients with stage IV tumors, there was not enough data to build the corresponding survival curves.

### 3.4. Functional Genetic and Protein Networks Structured by the Genes Comprising the 8-GS

To further analyze the molecular and biological profile of the genes structuring the 8-GS, we conducted functional and interaction-based analyses using the GeneMANIA and the STRING databases. In the GeneMANIA environment, we based our network analysis on weighting by molecular function-based gene ontology (GO).

**Figure 6:**
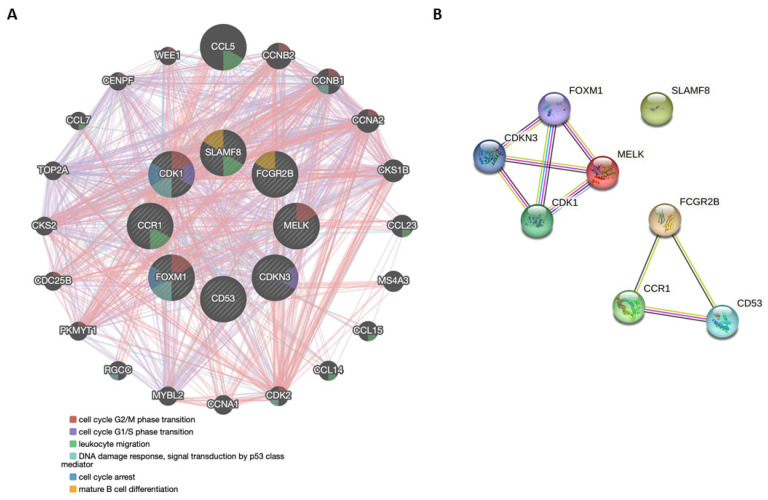
Functional network of the 8-GS. (**A**) The interaction network constructed by the 8-GS via the GeneMANIA database. The colored lines that connect the interrelated genes depict different types of interactions, including physical interactions (pink lines), co-expression (purple lines), co-localization (blue lines), and genetic interactions. The colors inside each node represent a distinct function attributed to the respective gene. The most commonly shared functions include (1) cell cycle G2/M phase transition; (2) cell cycle G1/S phase transition; (3) leukocyte migration; (4) DNA damage response, signal transduction by p53 class mediator; mature B-cell differentiation; (5) cell cycle arrest; and (6) mature B-cell differentiation. (**B**) Core interactions between the 8-GS gene products. The respective proteins were mapped in the protein-protein interaction (PPI) network using the STRING database. Nodes and edges represent proteins and their interactions, respectively. Filled, colored nodes signify the query proteins and first shell of interactors with known or predicted 3D structures. Known interactions, either extracted from curated databases or experimentally determined, are designated by turquoise and pink edges, respectively. Yellow edges show text-mining interactions; black edges indicate that the respective proteins are co-expressed. Purple edges interconnect proteins with sequence homology.

The most common pathways are related to cell-cycle promotion and regulation: cell-cycle G2 to M phase transition (*CDK1*, *MELK,* and *FOXM1*), G1 to S phase transition (*CDK1* and *CDKN3*), p53-based DNA damage response (*FOXM1* and *CDK1*), and cell cycle arrest (*FOXM1* and *CDK1*). *SLAMF8* and *CCR1* are implicated in leukocyte migration and in mature B cell differentiation (*SLAMF8* and *FCGR2B*) (Figure 6A). The interplay among the resulting network is composed of physical interactions (87.39%), co-expression (6.25%), co-localization (2.68%), genetic interactions (2.59%), and common pathways (1.08%). Regarding exclusively the genes of the 8-GS, physical interactions occur between the proteinic products of *CDK1* and *CDKN3*, and *CDK1* and *FOXM1* (Figure 6A; Pink lines). Most of the genes are co-expressed (Figure 6A; Purple lines); *FOXM1* and *MELK* are co-localized (Figure 6A; Blue lines); and genetic interactions have been reported for *CDK1* and *FOXM1*, as well as between *CD53* and *MELK* (Figure 6A; Green lines). Finally, *CDK1* and *FOXM1* are involved in the same signaling pathway(s) (Figure 6A; Light blue lines).

We also investigated the core interactions of the eight gene products by mapping them to the protein-protein interaction (PPI) network provided by the STRING database (Figure 6B). The analysis provided a proteinic network that could actually be subdivided into two: (i) MELK, FOXM1, CDKN3, and CDK1, and (ii) FCGR2B, CD53, and CCR1. SLAMF8 was not integrated into any sub-network. The entire network comprising the eight genes provides an average node degree—indicating the average number of edges per node—of 2.25, an average local clustering coefficient—representing the strength of the adjacent node interconnection and ranging between 0 and 1—of 0.875, and a *p*-value of PPI enrichment that indicates the significance of interactions equal to 2.42 × 10^6^. This means that there are a significant number of interactions among the respective proteins, which in turn implies that these proteins are at least partially biologically associated. As shown in Figure 6B, this is evident for the subgroup consisting of MELK, FOXM1, CDKN3, and CDK1, which would be anticipated since all of these proteins are involved in the regulation and promotion of the cell cycle. According to STRING-based local clustering, the above four gene products are part of a larger 45-protein network implicated both in mitotic cytokinesis and gastric cancer progression. GO analysis returned the following results: 6 out of the 8 proteins are regulators of phosphorylation (SLAMF8, CCR1, CDKN3, FOXM1, FCGR2B, and CDK1), 4 out of the 8 proteins (MELK, FOXM1, CDKN3, and CDK1) are involved in the mitotic cell cycle phase transition, and 3 out of the 8 (MELK, FOXM1, and CDK1) are involved in the mitotic G2/M transition. Text-mining interactions along with co-expression were revealed between all pairs of proteins in the two separate subgroups (MELK/FOXM1, MELK/CDKN3, MELK/CDK1, FOXM1/CDKN3, FOXM1/CDK1, CDKN3/CDK1, and FCGR2B/CCR1, FCGR2B/CD53, CCR1/CD53). Evidence of direct protein-protein interactions has been demonstrated for CDKN3 and CDK1 and for FOXM1 and CDK1. Finally, GSEA revealed that there is no universal hallmark under which all eight genes fall. However, 3 out of the 8 genes fall under the E2F targets hallmark, 2 genes fall under the allograft rejection hallmark, and finally, 2 genes fall under the G2M checkpoint hallmark and the spermatogenesis hallmark. These results are in accordance with the GeneMania and STRING results showing that cell proliferation and immune regulation are among the main functions of the identified genes.

## 4. Discussion

Although current progress on novel diagnostics and molecular prognosticators has significantly increased timely detection and allowed the prediction of post-treatment clinical outcomes in PCa patients, respectively, there is still a scarcity of established immune biomarkers for the prognosis of early-stage PCa patients’ survival rates. Therefore, the need for reliable, easily accessible immune-related molecular biomarkers that could be combined with clinically defined criteria, including PSA and Gleason score, to accurately predict PCa patients’ clinical outcomes is emerging. In the current study, we compared immune response-related gene expression in the peripheral blood of PCa patients with localized disease relative to healthy donors. We identified a gene signature comprising eight upregulated genes, variously related to the immune response and other critical cellular functions.

Four out of the eight genes comprising the 8-GS, namely *MELK*, *FOXM1*, *CDK1*, and *CDKN3*, are mainly implicated in cell cycle promotion and proliferation; consequently, aberrant expression of the respective gene products may lead to diverse degrees of malignant development and progression. Interestingly, although not among their principal functions, a growing body of evidence associates these genes primarily with tumor immune infiltrating lymphocytes and other immune cell populations and secondary with other immune responses. In vitro and in vivo studies have spotlighted MELK as a central driver of both cancer progression and relapse, thus highlighting its prominence as a therapeutic target [20]. *MELK* overexpression has been associated with intra-tumoral immune responses and immune cell infiltration, including pro-tumoral T helper type 2 cells and regulatory T cells [21]. Increased expression of *FOXM1* has been found to be capable of derailing proper anti-tumor immune responses through its direct binding to and subsequent upregulation of *PD-L1* [22]. *CDK1* upregulation has been associated with intra-tumoral immune alterations in a variety of human cancers, including high levels of expression of lymphocytes negatively regulating antitumor immunity [23], as well as Wnt/β-catenin activation [24,25,26], known to diminish STING activation followed by reduced priming of antitumor T cell immune responses. *CDKN3* overexpression is linked to high *MYC* expression and is associated with a poor prognosis. Upregulation of this gene has been recently reported to significantly correlate with hypomethylated promoter status, advanced T stage, metastasis, and aberrant antitumor immunity in cancer patients [27].

A second sub-network of gene products could be identified, composed of FCGR2B, CD53, and CCR1. FC gamma receptor IIB (*FCGR2B*) overexpression in hematopoietic progenitor cells triggers the generation and expansion of myeloid-derived suppressor cells (MDSC) that target cytotoxic CD8+ T cells, thus hampering the intrinsic anti-tumor immune response [28]. Equally, tumor- or therapeutically-triggered hypoxia in the tumor microenvironment (TME) may increase *FCGR2B* expression on mononuclear cells and macrophages, thus hindering their ability to phagocytose tumor cells [29]. CD53 is actively associated with NK and T-cell responses, mainly through regulating the activation and proliferation of these cell populations [30,31]. Notably, *CD53* depletion led to diminished CD4+ and CD8+ cell proliferation both in vitro and in vivo [32]. C-C chemokine receptor type 1 (CCR1) is a mediator of neutrophil, monocyte, and lymphocyte recruitment in sites of inflammation, and, along with its ligands, it has been linked to cancer cell survival, migration, and invasion [33,34]. Targeted *CCR1* downregulation was able to halt the metastatic expansion of colon cancer cells to the liver in vivo through the blockade of immature myeloid cell recruitment [35].

Finally, signaling lymphocytic activation molecule 8 (SLAMF8) is a transmembrane protein expressed by macrophages and suppresses their functions [36]. In patients with gastrointestinal (GI) tumors, increased serum levels of SLAMF8 bear high diagnostic significance [37], while its value for predicting response to immunotherapies has also been shown since immunohistochemistry analysis revealed enhanced CD8+ T cell infiltration in GI tumors with increased expression levels of *SLAMF8* taken from patients under anti-PD1 treatment [38].

Importantly, all of the above genes have proven prognostic and/or predictive value in various cancer types. MELK is widely considered an oncogenic kinase since it has been found overexpressed and associated with tumor growth, metastasis, recurrence, and ultimately a considerably poor prognosis in cancer patients, including those bearing tumors of the breast, lung, esophagus, and liver, among others [39], while its overexpression has been incriminated for the observed resistance to chemotherapy and radiotherapy [40,41]. FOXM1 upregulation is a poor prognostic factor in many solid tumors [42], and its therapeutic targeting is being exploited for the reinforcement of anti-cancer strategies. Likewise, in certain cancer types, including tumors of the breast, lung, and colon, high levels of CDK1 have also been associated with poor prognosis [43], while upregulation of CDKN3 has been underscored as an independent poor prognostic factor in ovarian cancer [44]. A profound biomarker utility of FCGR2B has been quite recently shown in patients with recurrent glioblastoma, in whom increased expression was correlated with decreased OS [45]. Accordingly, high levels of *CCR1* in patients with multiple myeloma have been linked to decreased overall survival [46], and increased levels of *SLAMF8* mRNA have been identified as a prognosticator of worse survival in patients with high-grade gliomas [47] and colorectal cancer [48]. Contrastingly, there is limited information on the prognostic role of *CD53* in cancer patients, with one study supporting that its upregulation may be associated with a disease-free survival benefit for patients with triple-negative breast cancer [49].

Based on the TCGA data of the PRAD cohort, increased intra-tumoral mRNA levels of the above 8 genes were individually associated with poor 5-year PFI in PCa patients. Importantly, the concomitant downregulation of all of these genes is being highlighted as a strong prognosticator of favorable PFI in PCa patients, even with Gleason scores above 8. The prognostic/predictive significance of the combination of the four genes implicated in cell proliferation, namely *MELK*, *FOXM1*, *CDKN3,* and *CDK1*, along with others, has already been described in several studies. A cluster of 10 overexpressed genes, including these four, was identified as a marker of resistance to immune checkpoint inhibition in patients with non-small cell lung cancer (NSCLC) [50] and renal cancer [51]. Similarly, an upregulated 22-GS, including mainly proliferation-related genes, was identified in biopsies of NSCLC patients lacking complete pathologic response to chemo-immunotherapy; among these, post-treatment upregulation of *MELK*, *FOXM1*, *CDKN3,* and *CDK1* was coupled with low pre-treatment PD-L1 levels and high densities of follicular T helper cells and M2 macrophages post-treatment [52]. Accordingly, the other four genes, namely *FCGR2B*, *CD53*, *CCR1,* and *SLAMF8,* were previously identified as integral components of a larger tumor associated macrophage-related signature with prognostic potential in patients with ovarian cancer [53]. In fact, there is a considerable amount of research on gene signatures, ranging from a few genes to large gene sets in size, with prominent prognostic value in distinct cancer types [54,55]. A remarkable finding of our analysis was the pan-cancer prognostic potential of the 8-GS; in this context, upregulation of the 8-GS was correlated with poor 5-year PFI and OS in cancer patients, even in those with early-stage tumors. These findings could be attributed to the vital role of these genes in critical cell and immune functions that are indiscriminately dysregulated in all tumors, contributing either to cancer development or progression and metastases.

In summary, our data presents an 8-GS with upregulated immune-related gene expression detected in the peripheral blood of PCa patients with localized disease. Our analyses from the TCGA database revealed that the expression of this 8-GS in the malignant tissue has been linked to poor prognosis in PCa patients at various stages of the disease and under different treatments. In addition, we could show that this unfavorable prognostic role for the 8-GS holds true for patients with a variety of cancer types. Combined with the increasing availability of public “omics” datasets, it is likely that this signature will be a valuable tool for researchers and oncologists to explore more precisely the molecular and cellular mechanisms that underlie tumor progression.

Intriguingly, a growing body of evidence associates these eight genes with a spectrum of immune responses, including regulation of tumor-infiltrating immune cell populations, and many studies have highlighted them as predictors of immunotherapy outcomes in distinct tumors, as described above [38,50,51,52,56]. Importantly, immune evasion predominates among the responsible mechanisms for PCa unresponsiveness to immunomodulatory therapeutic regimes [57,58], which is orchestrated by the presence of low numbers of infiltrating lymphocytes, high densities of intra-tumoral suppressive cells, and indolent antigen-presenting cells in the tumor microenvironment that deregulate the balance between the immune mechanisms structuring a robust anti-tumor immune response [59,60]. Given their observed immunosuppressive functions, concomitant inhibitory targeting of the upregulated genes composing the 8-GS could potentially enhance immunotherapy success rates and should be further exploited in therapeutic schemes for PCa patients.

The limitations of our study are mainly summarized in two distinct points: Firstly, our findings need to be confirmed in larger patient and healthy donor cohorts so that more sound conclusions can be drawn. Secondly, the absence of sufficient patients’ follow-up data did not allow us to correlate the observed upregulation of the 8-GS in the blood with the clinical outcome; for this reason, we investigated the effect of the overexpressed signature in tumor tissue specimens available from the TCGA database on the 5-year PFI of PCa patients. By validating our data in larger patient cohorts with localized disease and with longer clinical follow-up, we will be able to confirm this novel blood-based 8-GS as a valuable prognostic biomarker for early-stage PCa and, in this way, contribute to the design of more effective treatments by avoiding over-treatment and minimizing side effects. 

## 5. Conclusions

Overall, our study showcased a blood-based gene signature composed of eight genes with differential implications in the immune system and cell cycle regulation, which holds strong prognostic potential not only for PCa but also for all cancer patients, independent of the tumor type.

## Figures and Tables

**Table 1:** Clinicopathological and treatment characteristics of the enrolled prostate cancer (PCa) patients.

Characteristics of Patients with Localized PCa (*n* = 23)	
Age at diagnosis (years)	
Median	73
Range	53–81
PSA (ng/mL)	
Mean	18.74
Standard Deviation	20.85
Range	5.51–100.00
Gleason Score	
6	4 (17.4%)
7	11 (47.8%)
8	4 (17.4%)
9	4 (17.4%)
Mean (range)	7 (1)
T stage	
T1c	3 (13.0%)
T2a, T2b, T2c	12 (52.2%)
T3a, T3b	8 (34.8%)
Type of therapy received up to the time of blood sampling	
ADT	17 (73.9%)
RP + ADT	6 (26.1%)

ADT: androgen deprivation therapy; RP: radical prostatectomy.

## Data Availability

The data presented in this study are available upon reasonable request.

## Supplementary Materials

The following supporting information can be downloaded at: https://www.mdpi.com/article/10.3390/cancers15143697/s1. Figure S1: Expression levels of the genes comprising the eight-gene signature (8-GS) in the peripheral blood of 23 prostate cancer (PCa) patients grouped based on the Gleason score. Each graph corresponds to the expression levels of each single gene in PCa patients with a Gleason score below 8 (*n* = 15) vs. PCa patients with a Gleason score equal to or above 8. Each column shows the log2-transformed average mRNA levels of each gene normalized by Reads Per Million (RPM). Statistical analysis was performed using individual non-parametric Mann-Whitney (unpaired) tests. The error bars designate the average (Avg) values with a 95% confidence interval range. *p*-values are indicated for each graph.
