# Supplementary figures and images for: A Blood-Based Immune Gene Signature with Prognostic Significance in Localized Prostate Cancer
Source: Cancers (Basel). 2023 Jul 20;15(14):3697. doi: 10.3390/cancers15143697 (PMC10377824; doi:10.3390/cancers15143697)

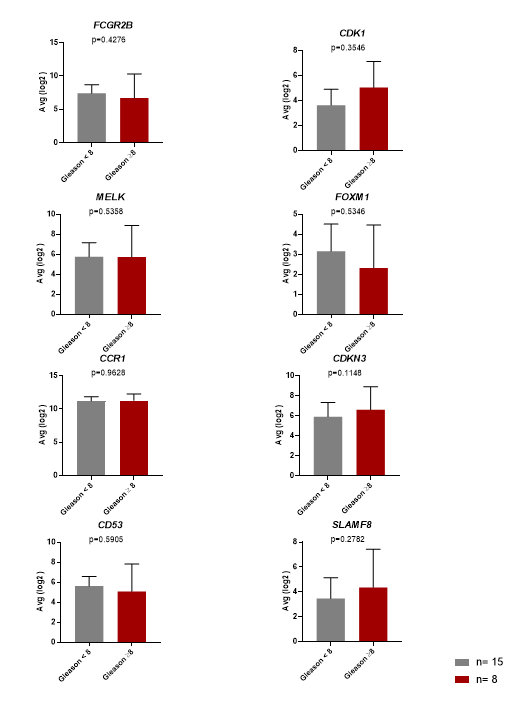

Supplement: Supplementary file 1 [file cancers-15-03697-s001.zip › Suppl. Figure S1.tif]
